# Development of Kovacs model for electrical conductivity of carbon nanofiber–polymer systems

**DOI:** 10.1038/s41598-022-26139-5

**Published:** 2023-01-02

**Authors:** Sajad Khalil Arjmandi, Jafar Khademzadeh Yeganeh, Yasser Zare, Kyong Yop Rhee

**Affiliations:** 1 Department of Polymer Engineering, Faculty of Engineering, Qom University of Technology, P.O. Box: 37195-1519, Qom, Iran; 2 Biomaterials and Tissue Engineering Research Group, Department of Interdisciplinary Technologies, Breast Cancer Research Center, Motamed Cancer Institute, ACECR, Tehran, Iran; 3 Department of Mechanical Engineering (BK21 Four), College of Engineering, Kyung Hee University, Yongin, Republic of Korea

**Keywords:** Engineering, Materials science

## Abstract

This study develops a model for electrical conductivity of polymer carbon nanofiber (CNF) nanocomposites (PCNFs), which includes two steps. In the first step, Kovacs model is developed to consider the CNF, interphase and tunneling regions as dissimilar zones in the system. In the second step, simple equations are expressed to estimate the resistances of interphase and tunnels, the volume fraction of CNF and percolation onset. Although some earlier models were proposed to predict the electrical conductivity of PCNFs, developing of Kovacs model causes a better understanding of the effects of main factors on the nanocomposite conductivity. The developed model is supported by logical influences of all factors on the conductivity and by experimented conductivity of several samples. The calculations show good accordance to the experimented data and all factors rationally manage the conductivity of PCNFs. The highest conductivity of PCNF is gained as 0.019 S/m at the lowest ranges of polymer tunnel resistivity (ρ = 500 Ω m) and tunneling distance (d = 2 nm), whereas the highest levels of these factors (ρ > 3000 Ω m and d > 6 nm) cannot cause a conductive sample. Also, high CNF volume fraction, poor waviness, long and thin CNF, low “k”, thick interphase, high CNF conduction, high percentage of percolated CNFs, low percolation onset and high interphase conductivity cause an outstanding conductivity in PCNF.

## Introduction

Various types of nanoparticles and polymer nanocomposites were studied in literature^1–15^. The combination of conductive carbon-based nanoparticles with an insulated polymer matrix causes the significant electrical conductivity at percolation onset^16–19^. One of the most important challenges for materials scientists is developing the low-weight conductive materials having enhanced conductivity^20^. Recently, carbon nanofiber (CNF) has been the favorable filler in developing of multi-role nanocomposites. Compared to conventional particles, CNFs are able to improve the mechanical and thermal properties as well as electrical conductivity of nanocomposites at very low volume fraction^21–27^. In comparison to other conductive carbon-based nanoparticles like carbon nanotubes (CNTs), CNFs have attracted interests in developing the conductive nanocomposites due to their low cost, since the high production cost of CNTs is a main limitation parameter. So, using CNF can cause an economic nanocomposites with outstanding properties^28,29^. A number of methods and modeling of PCNFs have been proposed in many papers, which includes modeling of their preparation, characterization, mechanical and thermal properties^30–32^. However, there has been no study that precisely concentrates on the simultaneous characterization of electrical properties of PCNFs. This motivated the current study, justifying further in-depth investigation of CNFs electrical effect on nanocomposites.

The electrical conductivity of PCNF is often evaluated by percolation theory^33^. Nanoparticles are separately dispersed inside polymeric matrix at low CNF concentrations, which result in poor conductivity of composites. Beyond a critical CNF volume fraction, nanoparticles are joined together and create a contiguous network. The percolation onset was primarily based on the shape and size of CNF^34,35^. Increasing aspect ratio (length per diameter) of the fillers causes a decrease in the percolation threshold^36^. The conductive nets can be formed more easily for the composites reinforced with CNFs with large aspect ratios, which grow the electron transferring. Besides, many literature works have demonstrated that the alignment and surface modification of the conductive phase can considerably affect the electrical conductivity of polymer composites^37–39^.

The electrical conductivity of PCNF is conducted by two mechanisms^40^. The conduction can be emanated from percolated networks and by tunneling effect between adjacent nanofibers. In other words, when the distance among conducting fillers is close enough, the tunneling conductivity will occur^41^. It is well recognized that the tunneling can play an important role in the conduction of PCNF^42,43^. Many developed or new models for the resistances of tunneling zones were suggested to guess the tunnels properties, since the electrical effectiveness of tunnels is outstanding in nanocomposites^38,44,45^. Furthermore, interphase regions are formed due to the robust interfacial linkage between nanofiber and matrix as well as the surface area of nanoparticles in nanocomposites^46–50^. The remarkable reinforcing effect of interphase zones was reported in the earlier studies^51–56^. In addition, the percolating structure of interphase layer was studied in several papers^57,58^. In fact, interphase regions are able to cause a percolating behavior at small volume fraction of CNF in nanocomposites, although this topic was ignored in the literature.

Kovacs et al.^59^ suggested a methodology for predicting the conductivity of CNT-containing nanocomposites, which considers the resistance of CNTs, CNT dimensions, tunneling impact and the weight percentage of CNT. In fact, their model was capable of relating the contact resistance between individual nanoparticles incorporated within a polymer matrix to the sample conductivity. Several experimental works have widely concentrated on original Kovacs model^60–64^. The published papers have expected to facilitate a further in-depth investigation and better understanding of polymeric nanocomposites reinforced by conductive fillers. In addition, the computed effective conductivity of nanocomposites and the magnitude of contact resistance between two adjacent fillers were evaluated. As discussed, many studies have described the electron tunneling as a significant mechanism in Kovacs model. However, there is no experimental and theoretical work that precisely highlights the effects of both interphase and tunnels zones on the conductivity of polymer composites in the Kovacs model. In the present work, Kovacs model is developed by assuming the tunneling and interphase regions, their intrinsic resistances and the volume fraction of CNF in the samples. So, the developed model is able to forecast the conductivity of PCNF by assuming the influences of tunneling and interphase regions in the networks. Parametric analyses and experimented outcomes are applied to indorse the developed model.

## Model development

A model was suggested by Kovacs et al.^59^ to determine the conductivity of CNT-based composites as:

1 $$\sigma = \frac{{lw_{f}^{2k + 1} }}{{2\pi R^{2} (R_{N} + R_{t} )}}$$

where “R” and “*l*” are the radius and length of nanofiller, respectively. “$w_{{f}}$” is the weight fraction of CNT and “$R_{\mathrm{N}}$” and “$R_{\mathrm{t}}$” are intrinsic resistances of CNTs and tunneling region, in that order. Also, “k” is an exponent. The experimental and theoretical studies calculated “2k + 1” between 2.7 and 5.3^59^. Interphase, CNF and the fractions of tunneling and interphase regions are able to handle the conductivity of PCNF^65^. However, Eq. (1) cannot assume the effects these factors on the conductivity. Based on these descriptions, a new methodology is developed to consider above parameters.

Percolation onset of CNF in PCNF is defined by using aspect ratio of CNF^66^ as:

2 $$\phi_{p} \approx \frac{1}{\alpha } \approx \frac{R}{l}$$

The interphase thickness is a layer surrounding CNF, which must be taken into consideration in Eq. (2), because the interphase regions are able to decline the percolation onset of CNF and accelerate the networking^67^. Therefore, final equation for the percolation threshold using Eq. (2) is expressed as:

3 $$\phi_{p} = \frac{15(R - 2t)}{l}$$

where “t” is interphase depth. CNFs are known to possess high aspect ratio, which results in the waviness in PCNF. In fact, their high aspect ratio causes a dramatic decrease in the efficiency of CNFs^68^. The waviness factor is presented by using an equivalent length ($l_{eq}$), which is reflected as the shortest length between two ends of a CNF as:

4 $$u = \frac{l}{{l_{eq} }}$$

where higher ranges of “u” indicate further curvature, whilst no waviness is achieved by u = 1.

Also, wavy CNF weakens its conductivity^69^. The effect of waviness on “$\sigma _{\mathrm{N}}$” as the nature conduction of CNF is expressed by:

5 $$\sigma_{Nw} = \frac{{\sigma_{N} }}{u}$$

CNF and its interphase depth are able to change the density of nets in the PCNF raising the conductivity. The total volume share of the interphase zone surrounding CNF^70^ is estimated as:

6 $$\phi_{i} = \phi_{f} \left(1 + \frac{t}{R} \right)^{2} - \phi_{f}$$

where “$\varphi _{{f}}$” is the volume fraction of CNF in PCNF. Moreover, CNF effective volume share is determined by CNF and its surrounding interphase regions, which is given by:

7 $$\phi_{eff} = \phi_{i} + \phi_{f} = \left(1 + \frac{t}{R}\right)^{2} \phi_{f}$$

Additionally, only some nanofibers are involved in the percolated network above percolation onset, whereas the remains are inactive in PCNF. The percentage of networked CNFs^71^ is evaluated as:

8 $$f = \frac{{\phi_{eff}^{1/3} - \phi_{p}^{1/3} }}{{1 - \phi_{p}^{1/3} }}$$

The volume fractions of both CNF and interphase networks can be calculated by using “*f*” as:

9 $$\phi_{N} = f\phi_{f}$$

10 $$\phi_{iN} = f(\phi_{eff} - \phi_{f} )$$

Moreover, the formation of tunneling areas occurs near the interphase regions. As a result, the volume share of tunneling regions is stated as:

11 $$\phi_{tN} = (\phi_{N} + \phi_{iN} ) \left(1 + \frac{d}{R + t}\right)^{2} - \phi_{N} - \phi_{iN}$$

where “d” is tunneling distance.

Now, the intrinsic resistances of CNF, interphase and tunneling regions are stated. CNF intrinsic resistivity is proposed by:

12 $$R_{N} = \frac{l}{{\pi R^{2} \sigma_{N} }}$$

Equation (12) considers the waviness by Eqs. (4) and (5) as:

13 $$R_{N} = \frac{{l_{eq} }}{{\pi R^{2} \sigma_{Nw} }} = \frac{l}{{\pi R^{2} \sigma_{N} }}$$

indicating that waviness cannot affect the intrinsic resistance of CNF, because “u” affects the “*l*” and “$\sigma _{\mathrm{N}}$” simultaneously. In addition, there is no equation to reflect the interphase conductivity in PCNF. It has been indicated that interphase conductivity as “$\sigma _{\mathrm{i}}$” is primarily based on the interactions between CNFs and matrix^41^, which is considered as 1000 S/m (in average) in our system. Based on these descriptions, the intrinsic interphase resistivity is presented as:

14 $$R_{i} = \frac{l}{{\sigma_{i} \pi t^{2} }}$$

The resistance of tunnels ($R\mathrm{t}$)^72^ is calculated by:

15 $$R_{t} = R_{1} + R_{2}$$

where “R_1_” and “R_2_” are the resistances of CNF and polymer matrix in the tunnels, correspondingly. However, “R_1_” is much lower than “R_2_” in calculations, which can be omitted from Eq. (15). So, the total resistance of tunneling regions is obtained by the resistance of polymer layer, which is calculated as a function of tunnel size and resistivity by:

16 $$R_{t} = R_{2} = \frac{\rho d}{s} = \frac{\rho d}{{3R^{2} }}$$

where “ρ” and “s” are polymer tunneling resistivity and contact area among nanofibers, respectively. Also, “s” is assumed to be $3R^{2}$ in terms of the end-to-body and end-to-end contacts among adjacent CNFs^72,73^. Therefore, the total resistance of a nanocomposite includes the resistances of CNF, interphase and tunneling regions as:

17 $$R_{c} = R_{N} + R_{i} + R_{t}$$

**Figure 1:**
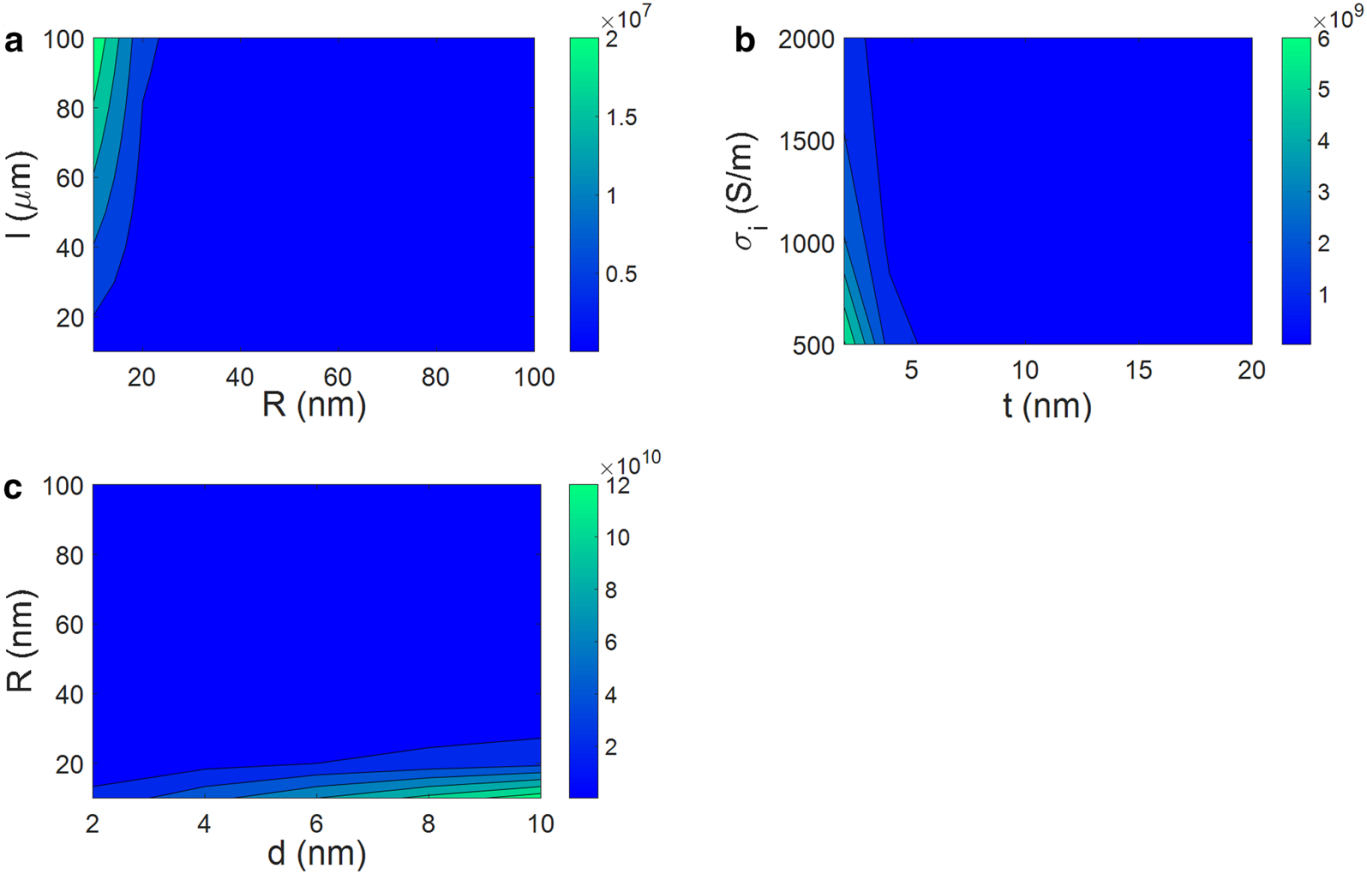
Impacts of main factors on the resistances of (**a**) CNF (Eq. 13), (**b**) interphase (Eq. 14), and (**c**) tunneling regions (Eq. 16).

However, all resistances have not the same effect on the conductivity of nanocomposite. According to Eq. (13), CNF resistance depends on its dimensions and conduction. Besides, the resistances of interphase and tunneling regions are primarily based on the interphase thickness and conduction of interphase as well as tunnel length and CNF radius. So, to understand the most and the least effectual resistances in the nanocomposites, the influences of parameters on the resistances of CNF, interphase and tunneling regions are plotted by contour diagrams in Fig. 1. As seen, CNF has the poorest resistance of $2 \times 10^{7}$ ohm in comparison to interphase resistivity ($6 \times 10^{9}$ ohm) and tunneling zones ($12 \times 10^{10}$ ohm). Therefore, CNF resistance can be removed from Eq. (17). As a result, the total resistance is calculated by:

18 $$R_{c} = R_{i} + R_{t}$$

The main equation (Eq. 1) is based on the weight fraction of nanoparticles in the system, whilst most models for conductivity of nanocomposites have considered the volume fraction of filler^40,68,74^. Thus, the volume portions of CNF, interphase and tunneling zones are used to develop Eq. (1) for conductivity of PCNF. By considering the roles of interphase and tunneling resistances, electrical conductivity of PCNF is expressed based on Eq. (1) as:

19 $$\sigma = \frac{{l(\phi_{N} + \phi_{iN} + \phi_{tN} )^{2k + 1} }}{{2\pi (R + t + d)^{2} R_{c} }}$$

reflecting the fractions of CNF, interphase and tunneling properties in the nanocomposites. In this paper, the developed equations are accurate when a CNF is randomly incorporated in the matrix. Based on our model, the random orientation of CNFs in the polymer matrix is significant, whereas the type of polymer matrix is unimportant.

**Figure 2:**
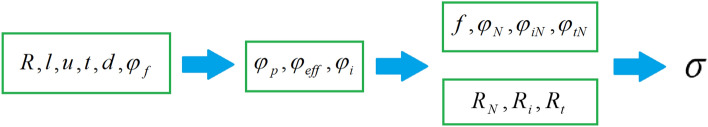
A diagram of developed model using Kovacs equation.

For a summary, Fig. 2 illustrates a diagram to show how the original Kovacs model has been modified in this study. By using the effective parameters of CNF and interphase, percolation onset, effective filler volume fraction and total volume fraction of interphase network are expressed. Furthermore, the volume fractions and instinct resistances of CNF, interphase and tunnels were applied to obtain the conductivity of samples by Eq. (19).

## Results and discussion

### Assessment of developed model using experimented results

The developed methodology is evaluated by the experimented outcomes of electrical conductivity for four samples from literature. In this manuscript, the samples were chosen as epoxy/CNF^75^, high density polyethylene (HDPE)/CNF^76^, epoxy/CNF^77^ and low density polyethylene (LDPE)/CNF^78^. The theoretical terms are expressed as CNF waviness, interphase thickness, tunneling distance, calculated percolation onset, the intrinsic tunneling resistance and “k”, whilst the experimental terms are CNF dimensions and experimental percolation onset.

The experimental and theoretical calculations of the mentioned parameters are given in Table 1. The theoretical approximations of percolation onset indicate a good agreement with the experimental data assuming interphase depth validating Eq. (3). The values of interphase thickness (t) are estimated in the range of 20 nm to 35 nm, which are reasonable, since the interphase depth changes at nanoscale below 100 nm. The presence of various interphase zones within the experimental samples express the dissimilar degrees of interfacial properties between CNF and polymer matrices. The thinnest (the poorest interfacial bonding) and the thickest (the strongest interfacial bonding) interphase are demonstrated in epoxy/CNF (No. 3) and HDPE/CNF samples, correspondingly.

**Table 1:** Experimental and theoretical values of factors for samples.

No.	Samples [ref.]	R (nm)	*l* (μm)	$\phi_{p}$ Exp.	u	t (nm)	d (nm)	$\phi_{p}$ Eq. (3)	ρ (Ω m)	k
1	Epoxy/CNF^75^	69	75	0.0075	1.3	20	4	0.0075	3000	0.0010
2	HDPE/CNF^76^	75	8	0.0100	1.2	35	4	0.0113	110	0.0001
3	Epoxy/CNF^77^	67	20	0.0029	1.1	32	2	0.0025	2000	0.0010
4	LDPE/CNF^78^	70	10	0.0243	1.2	28	7	0.0252	600	0.0010

**Figure 3:**
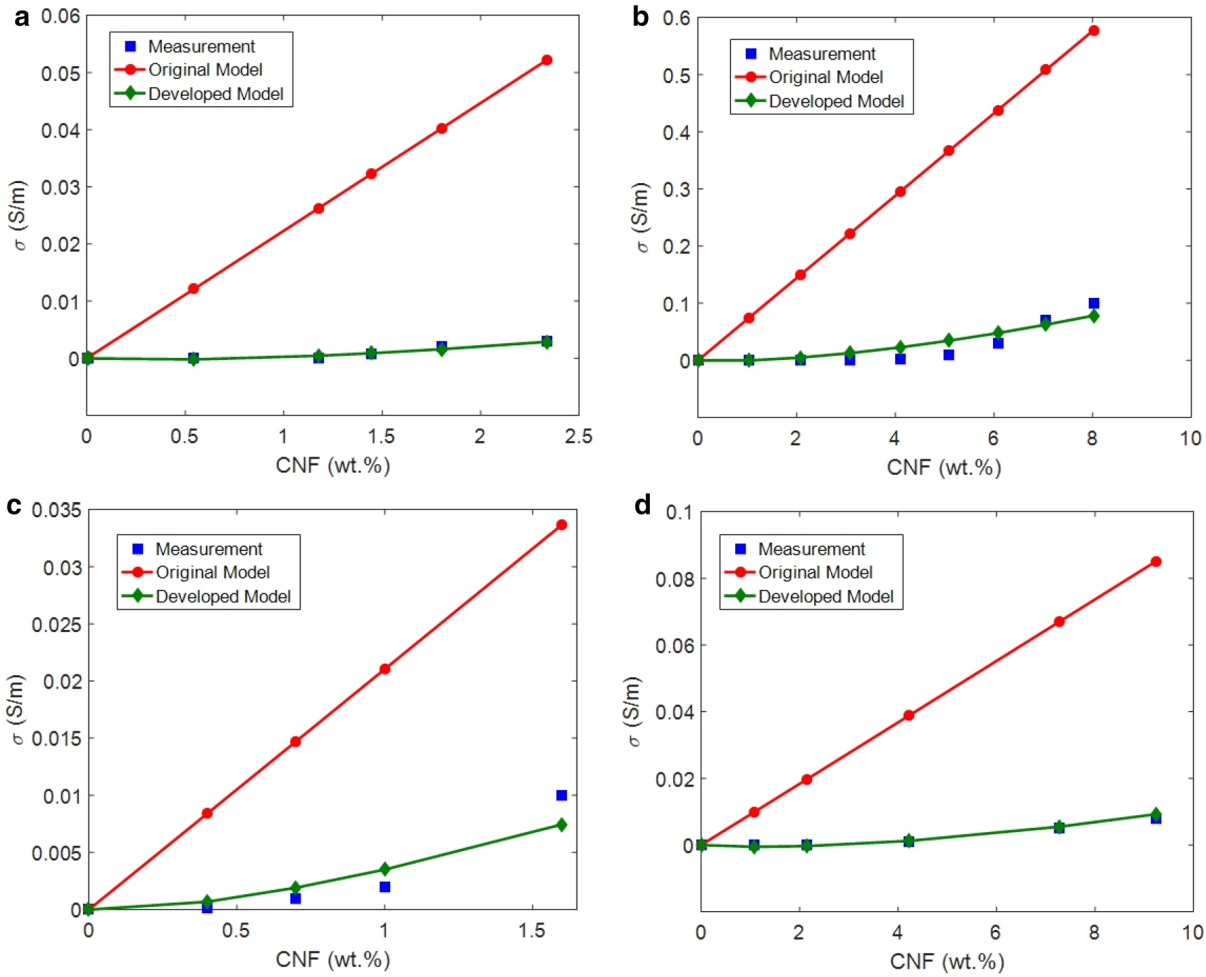
Experimental results and the calculations of conductivity by original model (Eqs. 1 and 19) for (**a**) epoxy/CNF^75^, (**b**) HDPE/CNF^76^, (**c**) epoxy/CNF^77^ and (**d**) LDPE/CNF^78^ samples.

To confirm the accuracy of the proposed model, the experimental data of conductivity and the calculations of original (Eq. 1) and developed models are presented in Fig. 3. Good agreements of Eq. (19) with experimented results are clearly evident approving the developed model, while the calculations of original model are higher than the experimental data. Furthermore, CNF waviness, tunneling distance, polymer tunneling resistivity as well as “k” can be calculated by the developed model. CNF waviness changes from 1.1 to 1.3 for the samples depending on the curvature of nanofiber in the systems. Tunneling distance is measured from 2 to 7 nm, which are meaningful in polymer nanocomposites, because it is less than 10 nm. Also, polymer tunneling resistivity is calculated from 110 to 3000 Ω m and “k” is obtained from 0.0001 to 0.001. The value of “k” was obtained between 0.0001 and 0.001 for the present samples as shown in Table 1. The lowest “k” is due to the highest conductivity of HDPE/CNF sample compared to others. In fact, the highest conductivity of HDPE/CNF sample causes the lowest “k” among the reported samples. All these data are reasonable for polymer nanocomposites verifying the developed model.

### Checkup of parameters

In this section, the impacts of main factors on the conductivity of PCNF are examined by the developed model. The typical values of parameters are considered as R = 50 nm, t = 10 nm, l = 50 µm, u = 1.3, d = 5 nm, $\varphi _{{f}}$ = 0.02, ρ = 4000 Ω m, k = 0.001 and $\sigma _{\mathrm{N}}$ = $10^{4}$ S/m (in average)^79–81^.

**Figure 4:**
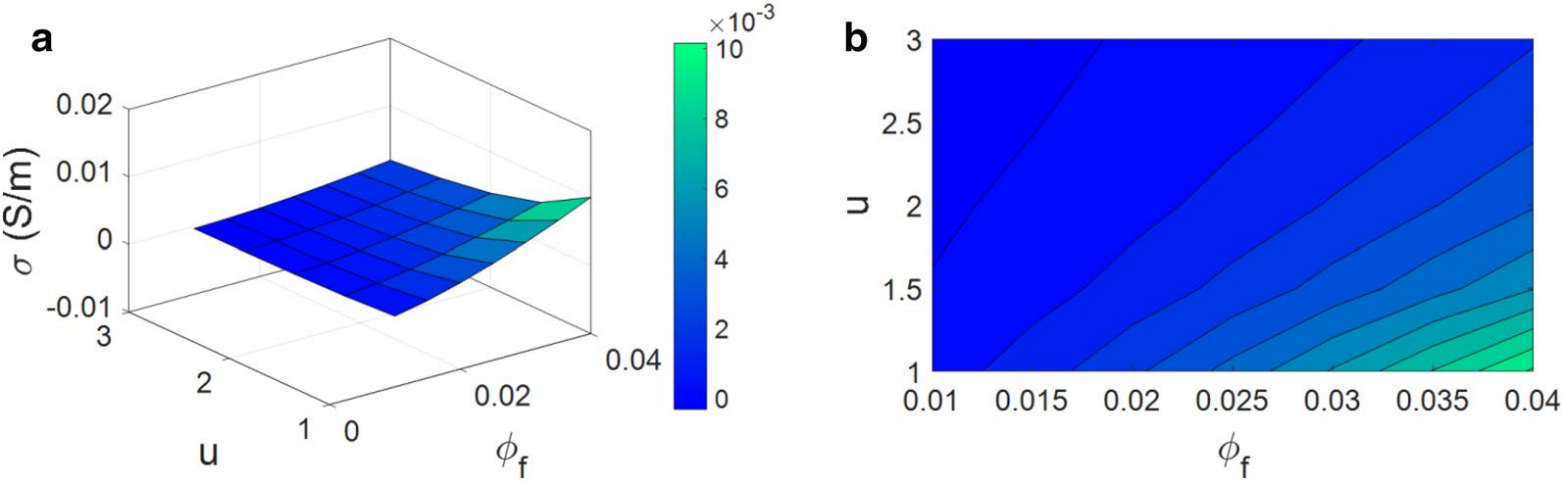
(**a**) 3D and (**b**) 2D plots for the conductivity as a function of “$\varphi _{{f}}$” and “u”.

The estimations of the conductivity related to “$\varphi _{{f}}$” and “u” are indicated in Fig. 4. The best outcome as 0.01 S/m is achieved by the highest level of “$\varphi _{{f}}$” and the lowest value of “u”, while poorer conductivity is obtained by lower ranges of CNF concentrations and higher levels of CNF curvature. As seen, the nanocomposite is insulated at $\varphi _{{f}}$ = 0.01 and u = 3 demonstrating the desirable efficiencies of high CNF concentration and low waviness in the conductivity of samples.

A higher level of CNF concentration can improve the conductivity of PCNF, because a considerable difference is witnessed between the conductions of CNF and polymer matrix. In other words, the conductivity of CNF was reported as $\sigma _{\mathrm{N}}$ = $10^{4}$ S/m, which is $10^{19}$ times greater than the conduction of polymer matrix^82^. However, a low CNF volume share does not lead to an increase in the conductivity of insulated matrix. Therefore, the concentration of CNF straightly determines the conductivity of nanocomposites, due to the higher range of CNF conductivity than that of polymer matrix^40^. Also, the curvature shortens the effective length of nanofibers. The waviness lowers the aspect ratio of CNFs and impedes the percolating behavior of nanoparticles in the networks^69^. In addition, the negative effect of the waviness on the conductivity of nanofiber is seen in Eq. (5). In fact, wavy nanoparticles cannot enhance the charge transferring in the system^83^. Hence, the reasonable influence of waviness on the conductivity is indicated by the suggested equations.

**Figure 5:**
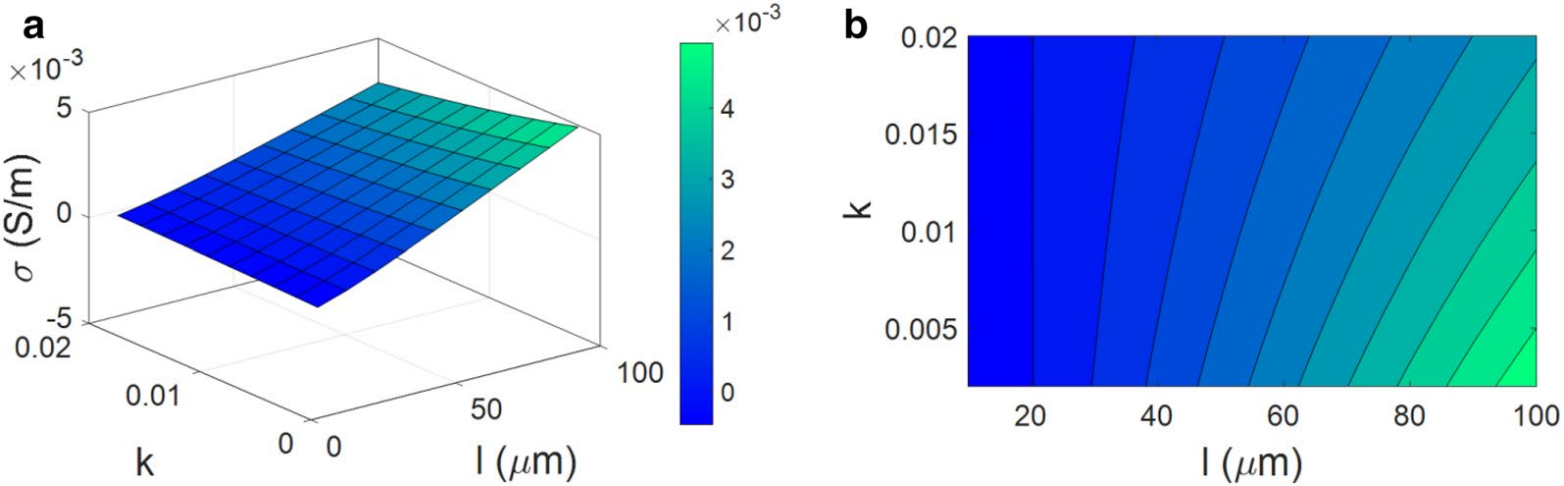
The influences of “l” and “k” on the conductivity by Eq. (19): (**a**) 3D and (**b**) 2D plots.

The impacts of “l” and “k” on the conductivity are exhibited in Fig. 5. The highest conductivity as 0.005 S/m is calculated at l = 100 µm and k = 0.002, whilst the poorest conductivity as 0 is observed at l < 20 µm at all levels of “k”. In fact, the most desirable conductivity is achieved at the highest level of “l” and the lowest range of “k”, but the lower values of CNF length and higher levels of “k” decrease the conductivity of nanocomposite.

CNF length controls the percolation onset (Eq. 3) and the percentage of networked CNF (Eq. 8). In fact, larger nanofibers positively decline the percolation onset and produce larger nets in PCNF, which efficiently transfer more charges among nanoparticles^84^. Conclusively, longer CNFs cause a confident raise in the density of networks enhancing the conductivity. In addition, a low “k” can considerably change the conductivity, due to its inciting role in the effectiveness of interphase, tunneling areas and filler in PCNF (see Eq. 19). However, a high level of “k” is not able to improve the conductivity of PCNF, because it weakens the conducting efficacies of CNF and interphase. So, the impacts of “l” and “k” on the conductivity are correctly exhibited by the developed model.

**Figure 6:**
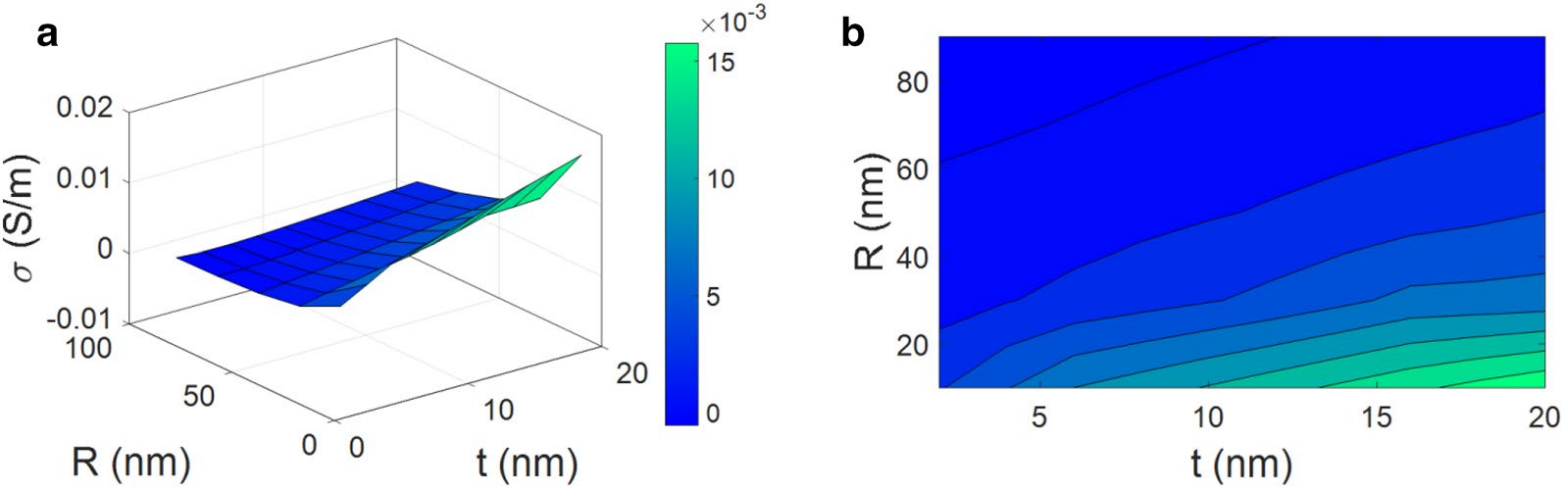
The stimuli of “t” and “R” on the conductivity by (**a**) 3D and (**b**) contour illustrations.

Figure 6 depicts the influences of “t” and “R” on the conductivity. The best result is obtained as 0.016 S/m with t = 20 nm and R = 10 nm. Therefore, a thick interphase layer and a thin CNF improve the conductivity significantly, but a thin interphase and a high level of CNF radius insignificantly improve the conductivity. t = 2 nm and R = 90 nm cannot grow the conductivity mentioning the undesirable effects of very thin interphase and too thick CNF on the conductivity.

A thick interphase layer surrounding CNF quickens the percolation (Eq. 3) and broadens the network of nanofibers based on Eq. (8). In other words, the distance between CNFs is decreased by the thicker interphase, thereby improving the conductivity. Additionally, the density and dimensions of nets can be increased by the participation of deep interphase regions^85^. Generally, a potential situation for enhancing the conductivity in PCNF is provided by the interphase regions around nanoparticles, which shows that a thick interphase improves the effective conduction of CNFs. Thus, the interphase depth correctly handles the conductivity by the developed model.

The percolation threshold can be shifted to low filler concentration by thin CNFs. Furthermore, the lower levels of CNF radius widen the interphase regions resulting in an enhancement of effective filler volume share in PCNF. Therefore, thin nanofibers produce the big networks, which cause a significant raise in conductivity. Also, it is evident from Eq. (16) that thin CNFs generate a low tunneling resistance providing a high conductivity. On the other hand, thick CNFs harmfully affect the percolation level, weaken the efficiency of networks and enhance the tunneling resistance causing a poor conductivity^68,86^. So, this explanation confirms the impact of CNF radius on the conductivity of PCNF using the developed model.

**Figure 7:**
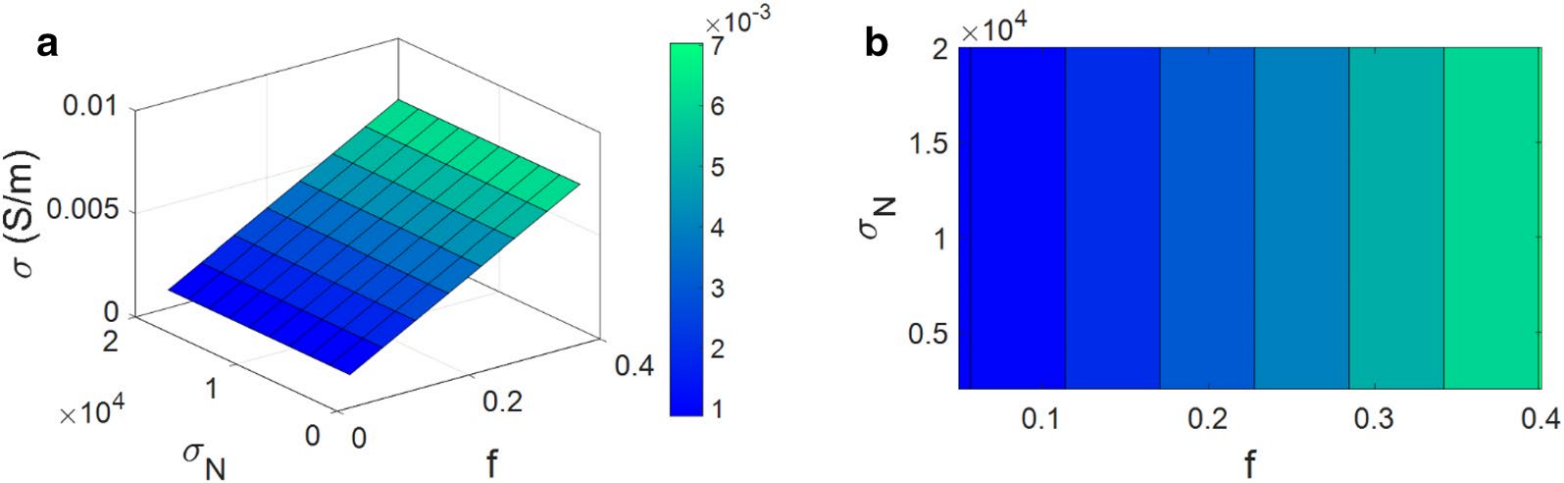
Effects of “*f*” and “$\sigma _{\mathrm{N}}$” on the conductivity by (**a**) 3D and (**b**) 2D intrigues.

Figure 7 displays the conductivity as a function of “*f*” and “$\sigma _{\mathrm{N}}$”. *f* = 0.4 causes the highest level of conductivity as 0.007 S/m at various levels of “$\sigma _{\mathrm{N}}$”, which shows that the best conductivity is obtained at the highest value of network fraction. However, the conductivity diminishes to about 0.001 S/m at *f* = 0.06 and various ranges of “$\sigma _{\mathrm{N}}$”. Therefore, the network fraction directly controls the conductivity, which should be maximized to reach a superior conductivity. Nevertheless, CNF conduction cannot control the conductivity.

“*f*” as the percentage of percolated nanofibers involved in the networks above percolation onset directly handles the density and size of CNF networks^71,82^. In fact, dense and big networks can be formed in PCNF when a higher number of nanoparticles are included in the networks. Obviously, a higher “*f*” facilitates the charge transferring among nanofibers in the percolated nets. However, a lower value of “*f*” indicates that more CNFs are dispersed in PCNF, which produce a smaller network. Accordingly, “*f*” directly manages the conductivity of samples approving the developed model. Although a higher “*f*” causes a higher conductivity, CNF conductivity cannot affect the conductivity. In fact, the percentage of percolated CNF directly controls the conductivity, but CNF conduction is ineffective. The conductivity of CNF is much greater than the conductivity of polymer medium, which largely minimizes the “R_N_” (Eq. 13). However, “R_N_” was removed from the total resistance of components (Eq. 18). This means that CNF cannot cause a resistance against the electrons and thus CNF conductivity cannot change the conductivity of PCNF. Accordingly, the developed model correctly forecasts the dependence of nanocomposite conductivity on the CNF conductivity.

**Figure 8:**
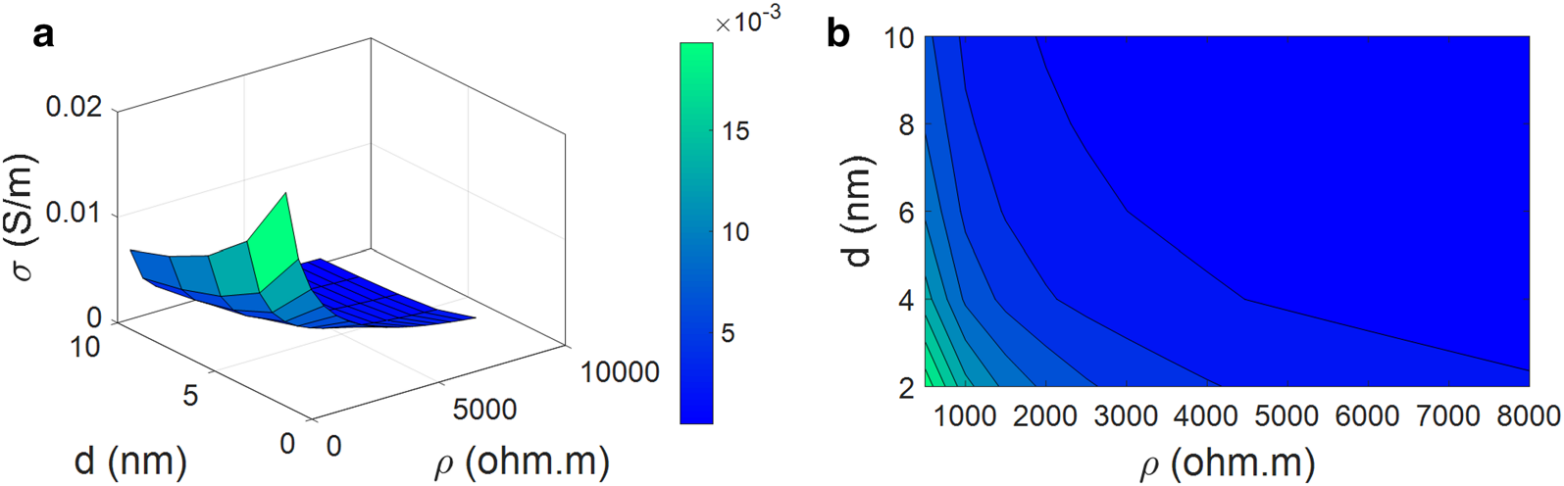
The various ranges of calculated conductivity at different levels of “ρ” and “d”: (**a**) 3D and (**b**) 2D diagrams.

The conductivity at different values of “ρ” and “d” is also exhibited in Fig. 8. An extraordinary conductivity as 0.019 S/m is achieved at the lowest levels of these factors, whereas non-conductivity is approximately introduced by the highest ranges of both “ρ” (> 3000 Ω m) and “d” (> 6 nm). Conclusively, a low polymer tunneling resistivity and a short tunneling distance are needed to acquire a conductive sample, although high tunneling resistivity and big tunnels cannot improve the conductivity.

Based on Eq. (16), a poor tunneling resistivity and short tunneling distance decrease the intrinsic tunneling resistance, which accelerates the electron transferring within the tunneling areas. On the other hand, higher tunneling resistivity and larger tunneling gaps enhance the resistance of tunneling region resulting in poorer charge transferring among nanofibers. In fact, the electron transferring between nanofibers is primarily based on the length between the surfaces of two adjacent CNFs^72^. However, a larger gap between CNFs cannot create the tunneling conductivity. Accordingly, the tunneling distance and resistivity inversely affect the conductivity. Certainly, the tunneling mechanism is controlled by the tunneling resistivity of polymer film and the separation distance between nanofibers^45^. So, the proposed model properly depicts the main roles of these factors in the conductivity.

**Figure 9:**
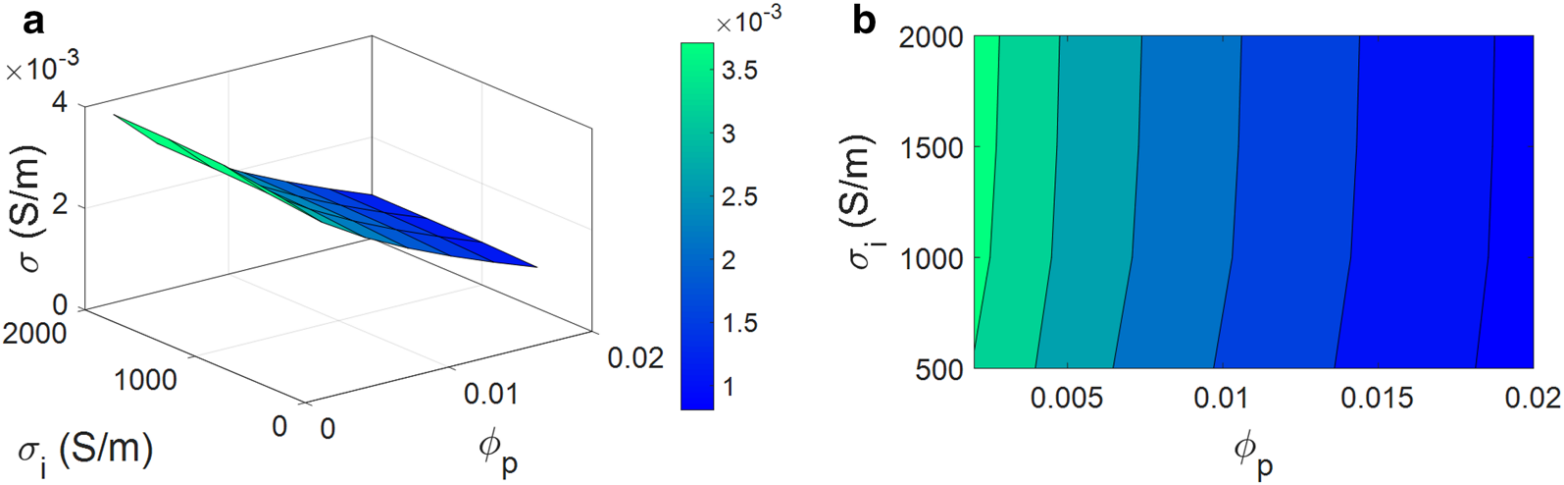
Theoretical conductivity by “$\varphi _{\mathrm{P}}$” and “$\sigma_{ \mathrm{i}}$”: (**a**) 3D and (**b**) 2D illustrations.

Figure 9 presents the roles of “$\varphi _{\mathrm{P}}$” and “$\sigma _{\mathrm{i}}$” in the conductivity. The conductivity is enhanced to maximum 0.0037 S/m by the low ranges of percolation onset and high values of interphase conduction ($\varphi _{\mathrm{P}}$= 0.002 and $\sigma _{\mathrm{i}}$ = 2000 S/m), whilst a high “$\varphi _{\mathrm{P}}$” and low “$\sigma_{ \mathrm{i}}$” produce a low conductivity. Also, $\varphi _{\mathrm{P}}$> 0.018 causes a poor conductivity (near to 0) at all ranges of “$\sigma _{\mathrm{i}}$”. Thus, it is crucial to lessen the percolation onset and increase the conductivity of interphase regions in order to grow the conductivity of system.

According to Eq. (8), low percolation onset raises the percentage of CNF in the nets, which positively manage the conductivity, because the larger nets can transfer more electrons in PCNF. However, higher levels of percolation onset lessen the network size declining the conductivity. So, a suitable relationship between percolation threshold and conductivity is established by the developed model. The same relation was reported between the mechanical percolation and the mechanical characteristics of nanocomposites^87^. Also, Eq. (14) has shown an inverse relation between the resistance of interphase regions and its conductivity, which indicates that a higher interphase conductivity produces a lower resistance of interphase. Indeed, higher interphase conductivity considerably reduces the total resistance increasing the conductivity of PCNF. The developed model donates a reasonable result, because a higher level of interphase conductivity shows the presence of highly-conductive interphase in PCNF. Conclusively, interphase conductivity straightly affects the conductivity of composites confirming the present model.

## Conclusions

Based on Kovacs equation, a new model was suggested to guess the electrical conductivity of PCNFs considering interphase, tunnels and CNF parts. Also, simple equations were applied to express the resistances and volume fractions of the mentioned phases in the networks. The proposed model successfully calculated the conductivity for various samples. Additionally, the conductivity was reasonably handled by all factors. The conductivity changed from 0 to 0.019 S/m at various ranges of all examined factors. The percolation onset was shown to be in the range of 0.0025 to 0.0252 based on our calculations, which presented a good accordance with the experimental data. A lower CNF waviness, higher filler concentration, longer nanofibers, lower “k”, thicker interphase, thinner CNF, higher fraction of networked CNF, less percolation onset, more interphase conductivity, poorer polymer tunneling resistivity and shorter tunneling distance produced a more conductivity, but the nature conduction of nanofiber was ineffective. It was calculated that the nanocomposite is insulated at $\phi_{f}$ = 0.01, $u$ = 3, l < 20 µm, t = 2 nm, R = 100 nm, ρ > 3000 Ω m and d > 6 nm. Amongst the examined factors, both polymer tunneling resistivity and tunneling distance pointedly influenced the conductivity from 0 to 0.019 S/m. It is expected that the developed model can be applied by researchers in future studies on PCNF. Since all effective factors were meaningful and determinate, the developed model presents a comprehensive methodology for the prediction and optimization of conductivity in PCNF.

## Data Availability

The data that support the findings of this study are available on a request from corresponding author.
